# Critical Care Nurses' Adherence to Ethical Codes and Its Association with Spiritual Well-Being and Moral Sensitivity

**DOI:** 10.1155/2023/8248948

**Published:** 2023-05-08

**Authors:** Marzieh Momennasab, Zohreh Homayoon, Camellia Torabizadeh

**Affiliations:** ^1^Department of Nursing, School of Nursing and Midwifery, Shiraz University of Medical Sciences, Shiraz, Iran; ^2^Student Research Committee, School of Nursing and Midwifery, Shiraz University of Medical Sciences, Shiraz, Iran; ^3^Community Based Psychiatric Care Research Center, Department of Nursing, School of Nursing and Midwifery, Shiraz University of Medical Sciences, Shiraz, Iran

## Abstract

**Background:**

Adherence to ethical codes is a major pillar of nursing care that is affected by various factors. Identifying these factors can lead to better ethical performance. The present study was conducted to determine critical care nurses' adherence to ethical codes and its association with spiritual well-being (SWB) and moral sensitivity (MS).

**Methods:**

In this descriptive-correlational study, data were collected using the moral sensitivity questionnaire (MSQ) by Lützén et al., Paloutzian and Ellison's spiritual well-being scale (SWBS), and the adherence to ethical codes questionnaire. The study was conducted on 298 nurses working in critical care units of hospitals affiliated with Shiraz University of Medical Sciences in southern Iran in 2019. This study was examined and approved by the Ethics Committee of Shiraz University of Medical Sciences.

**Results:**

The majority of the participants were female (76.2%) and single (60.1%), with a mean age of 30.69 ± 5.74 years. The mean scores of adherence to ethical codes, SWB, and MS were 64.06 (good), 91.94 (moderate), and 134.08 (moderate), respectively. Adherence to ethical codes had a positive correlation with the total score of SWB (*P* < 0.001, *r* = 0.25) and MS (*P* < 0.001, *r* = 0.27). A positive correlation was also observed between MS and SWB (*P* < 0.001, *r* = 0.41). Meanwhile, MS (*β* = 0.21) had a greater effect than SWB (*β* = 0.157) on adherence to ethical codes.

**Conclusion:**

Critical care nurses showed a good adherence to ethical codes. MS and SWB also positively affected their adherence to ethical codes. Nursing managers can use these findings to devise plans for the promotion of MS and SWB in nurses and thus help improve their ethical performance.

## 1. Introduction

Nursing ethics was formed to find the most appropriate way of providing nursing care [1]. Nurses must actively and continually adhere to the codes of ethics of the nursing profession so that they can provide optimal patient care in the course of clinical decision-making. Nurses must observe professional codes of ethics in their workplace so that they can make the best decisions about their patients when faced with challenges requiring clinical and ethical decision-making [2, 3].

Ethical issues are more common in critical care units [4], and critical care nurses are at high risk for moral distress [5]. Due to the particular features of critical care units, observing the patients' rights and making ethical decisions require greater attention [6], as patients in critical care units are in critical conditions, and it is thus necessary to be sensitive to observing their rights [7]. So, an ethical viewpoint should be developed in these settings [8], and more information must be obtained about healthcare providers' ethical performance and its related variables and concepts.

Despite the 2011 legislation on nursing codes of ethics in Iran, encompassing 12 values and 71 professional codes in five sections [9], the studies conducted in Iran have reported nurses' adherence to ethical codes as poor to good [10–12]. Many variables can affect the level of adherence to ethical codes, including the demographic and professional characteristics of the nurses [11]. Certain ethical and spiritual characteristics of nurses appear to affect their ethical performance and commitment to observing ethical principles in patient care [13]. One such factor is moral Ssensitivity (MS). MS is an attribute that enables nurses to identify ethical dilemmas, have a proper sensory and intellectual understanding of the situation, properly recognize the correct ethical action, and ultimately adopt the best decisions based on ethical considerations [14]. A study conducted by Kim et al. in Korea showed a positive and significant relationship between moral sensitivity and the use of ethical codes by nurses [15]. Although no studies were found on the relationship between these two variables in Iran, the results of several studies have shown a relationship between nurses' moral sensitivity and e ethical performance [15–17].

Another factor that may have a relationship with adherence to ethical codes is spiritual well-being (SWB), which is understood differently in different societies [18]. Since spirituality (in its different forms) is currently a major concern of mankind, it is highly worthy of incorporation into human and organizational behavior models and literature [19]. Spirituality in the workplace is so important that it should be regarded as the core of healthcare provision [20]. The review of the theoretical principles and research background show that spirituality has a relationship with social responsibility [21] and ethical issues in organizations [22].

SWB is “a sense of harmonious interconnectedness between self, others/nature, and the ultimate other that exists throughout and beyond time and space and can be achieved through a dynamic and integrative growth process leading to the realization of the ultimate purpose and meaning of life” [23]. Since moral principles are considered valuable in all religions and faiths [24, 25], nurses' spiritual well-being can be expected to affect their adherence to ethical codes [25].

Of the many studies conducted on spirituality and ethics in nursing, most have described the current state of affairs, and the relationship between these concepts and nurses' performance has received less attention. The review of the literature did not show any studies specifically conducted on the relationship of MS and SWB with adherence to ethical codes. In addition, most studies have assessed these concepts separately, and no studies could be found that had addressed the manifold relationships between these three variables. The present study was therefore designed and conducted to determine the adherence of critical care nurses to ethical codes and its relationship with MS and SWB.

## 2. Methods

### 2.1. Design

This study was a descriptive-correlational research. It adheres to the STORBE guidelines.

### 2.2. Participants and Setting

A total of 298 nurses participated in this study. They worked in adult critical care units of six hospitals affiliated with Shiraz University of Medical Sciences, southern Iran. The study inclusion criteria were as follows: a bachelor's degree and higher qualifications and a minimum of three months' work experience in adult critical care units. Incomplete questionnaires and desire to withdraw from the study were considered exclusion criteria.

Based on a study by Mahdiyoun et al. [26], *α* = 0.95, and *r* = 0.3, the sample size was determined as 154, which was increased to 170 to take account of a potential sample loss of 10%. Meanwhile, to increase the study's rigor, 305 adult critical care nurses were assessed. Seven incomplete questionnaires were removed, and data analysis was done on 298 of them. Multistage sampling was carried out, and a quota was assigned to each hospital in proportion to its number of eligible nurses. Then, samples were selected from each ward by systematic random sampling.

### 2.3. Data Collection

Data were collected using a demographic questionnaire (inquiring about personal details such as gender, age, work experience, academic qualification, and job position) and the following three questionnaires: the adherence to ethical codes questionnaire, the moral sensitivity questionnaire (MSQ), and the spiritual well-being scale (SWBS).

The 26-item adherence to ethical codes questionnaire was derived from the “Nurses and Practice” section of the Iranian nursing code of ethics. Each item is scored from 0 to 3 (from “never” = 0 to “always” = 3), with minimum and maximum scores of 0 and 78. The validity and reliability of this questionnaire have been previously confirmed in the study by Momennasab et al. [27], and its content validity was also confirmed based on the views of five nursing professors. Its reliability was confirmed by a test-retest with a correlation coefficient of 0.9 (20 nurses and 20 nurse managers completed the questionnaires in two stages with an interval of 2 weeks). In another study, the content validity and reliability of this tool were confirmed [28].

The MSQ was developed by Lützén et al. in Sweden and has been used after validation in various countries on different continents, including Iran. This 28-item questionnaire measures the moral sensitivity of nurses based on a 7-point Likert scale, from “totally agree” (7 points) to “totally disagree” (1 point), making for a total score of 28 to 196. This questionnaire has six dimensions, including using moral notions, benevolence, respect for the patient's autonomy, knowing patient communication skills, adherence to the law, and moral conflict experience. A score of 28 to 84 in this questionnaire was taken to indicate a poor, 85 to 141 moderate, and 142 to 196 high level of moral sensitivity [29]. The validity and reliability of this questionnaire have been confirmed in previous studies in critical care settings [30]. The reliability of the Farsi version of this questionnaire was confirmed by Hariri et al. with a Cronbach's alpha of 75% [31] and another study in critical care settings [32].

The SWBS was designed and introduced by Paloutzian and Ellison in 1982 [33]. This 20-item scale measures the subject's religious health with ten items and existential health with the next ten items. Scoring is based on a Likert scale from 1 to 6 points (“totally disagree” = 1 to “totally agree” = 6), with minimum and maximum scores of 20 and 120. A score of 20 to 40 was taken to indicate a poor SWB, 41 to 99 moderate, and 100 to 120 good SWB [34]. This scale has been used in many studies in different countries with confirmed validity and reliability. For the original version, Cronbach's alpha coefficient was reported as 0.93 and the test-retest reliability coefficient as 0.93 [33]. The content validity and reliability of the Farsi version were confirmed in other studies with Cronbach's alpha coefficients of 0.89 [35] and 0.91 [36]. In another study on acute myocardial infarction patients, the face, content, and construct validity, as well as the reliability of the Persian version of this scale, were confirmed [37].

In the present study, the researcher visited the adult critical care units of hospitals affiliated with Shiraz University of Medical Sciences over different work shifts and selected eligible nurses by systematic random sampling. The questionnaires were completed through the self-reporting method. The adherence to ethical codes questionnaire was completed by the nurses as a self-report and also separately by the head nurses of each ward, concerning the nurses under her management, and the mean value of the two scores was recorded as the nurse's adherence to ethical codes. Data were collected over six months, from March to August 2019, and 298 questionnaires were ultimately analyzed.

### 2.4. Data Analysis

The collected and encoded data were entered into and analyzed in SPSS-22. The normal distribution of the data was confirmed using the Kolmogorov–Smirnov test. The correlation between two quantitative factors was assessed by Pearson's correlation test, and the linear relationship between the continuous variables was assessed by simple linear regression. The independent *t*-test was used to compare the two groups in terms of the mean value of a quantitative factor. A one-way analysis of variance (ANOVA) was used to compare the three groups in terms of the mean value of a quantitative score. The level of statistical significance was set at 0.05 for all the tests.

### 2.5. Ethical Considerations

The study was approved by the Research Ethics Committee of Shiraz University of Medical Sciences, Shiraz, Iran (code: IR.SUMS.REC.1397.872.). All participants signed an informed consent after receiving information about the goals of the research, methodology, and confidentiality of any disclosed information. They were also assured that their participation had no effect on their professional status.

## 3. Results

A total of 29 nurses with a mean age of 30.69 ± 5.74 years (ranging from 23 to 50 years) and a mean work experience of 6.58 ± 5.94 years (ranging from 6 months to 26 years) participated in this study. The majority of the participants were female (76.2%) and single (60.1%), had no child (79.2%), had a bachelor's degree (95.6%), and graduated from public universities (68.5%) (Table 1).

Their mean score of adherence to ethical codes was 64.06 ± 7.11, which indicated a good level, given that the questionnaire score ranged from 0 to 78 points. This variable had no significant relationship with any of the personal and professional attributes of the nurses (Table 1).

The mean score of nurses' SWB was 91.94 ± 11.82, and the majority of them (74.2%, *n* = 221) were in the moderate range in this variable. The mean scores of religious and existential health were 47.62 ± 5.82 and 44.32 ± 6.84, respectively. The mean score of MS was 134.08 ± 9.05 in the participants, and the majority of them were in the moderate range (79.5%, *n* = 237).

Pearson's correlation test results showed a significant positive relationship between adherence to ethical codes and the total SWB score plus the scores of its dimensions (*P* < 0.05). Therefore, the mean score of nurses' adherence to ethical codes increases significantly as their mean scores of SWB and its dimensions increase (Table 2).

Pearson's correlation test showed that MS has a significant positive relationship with nurses' adherence to ethical codes (*r* = 0.27). Moreover, the mean score of nurses' adherence to ethical codes had a significant positive correlation with dimensions, including the use of moral notions, benevolence, respect for the patient's autonomy, and adherence to the law (*P* < 0.05), but a significant negative correlation with the experience of moral conflicts (*r* = −0.13) (*P* = 0.03), and no significant relationship with knowing patient communication skills (*P* > 0.05) (Table 3).

Pearson's correlation test also showed a significant positive relationship between moral sensitivity and spiritual health (*r* = 0.41). The total score of spiritual health and the two dimensions of religious and existential health had significant positive correlations with dimensions, including the use of moral notions, benevolence, adherence to the law, and the total score of moral sensitivity (*P* < 0.05).

The multivariate regression test showed a significant relationship between the mean scores of SWB, MS, and adherence to ethical codes in the nurses (*P* < 0.05). When the MS was kept constant, a 1-unit change in the SWB score affected the adherence to the ethical codes score by 0.094. Moreover, when the SWB was kept constant, adherence to the ethical codes score increased by 0.162 per 1-unit change in the MS score. Adherence to ethical codes is therefore more influenced by MS (*β* = 0.21) than by SWB (*β* = 0.157) (Table 4).

## 4. Discussion

According to the results, nurses working in critical care units had a good level of adherence to ethical codes. Other studies conducted in Iran have also shown that nurses have a favorable level of ethical performance and compliance with ethical codes in providing care [11, 26, 38]. Nonetheless, other studies have reported moderate [39] and even poor levels of adherence for some codes [10, 40]. Nurses' ethical performance appears to have improved according to recent studies compared to earlier ones, which could be due to hospitals' greater emphasis on observing patients' rights and the moral improvement of their personnel in line with the accreditation programs stressed by the Ministry of Health and Medical Education. As a matter of fact, hospitals' evaluation is partly designed based on adherence to ethical principles. Accordingly, in recent years, patients have been extensively notified of the various routes through which they can report their complaints about the performance of various units in each hospital by phone, email, or website. Moreover, ongoing continuous education programs about nursing ethics are provided in hospitals, and nursing ethics courses have been introduced to bachelor's and master's nursing curricula with codes of ethics as one of their main topics.

Observing ethical codes is more crucial in critical care units, where patients are either in critical conditions or unconscious and thus highly vulnerable, than in other wards [6]. Mere knowledge of ethical codes does not ensure their practice, and the individual must be sensitive enough to practice these codes [38].

According to the results, the participating nurses had moderate SWB scores. Critical care nurses also had moderate levels of SWB in some other studies [41, 42]. SWB refers to a sense of acceptance, positive feelings, morality, and a positive mutual relationship with an Almighty power, others, and the self, and is achieved through a dynamic and integrative cognitive, emotional, and interactive process [43].

According to the results, the participating nurses had moderate levels of MS, which agrees with the results of other studies. In studies conducted by Kim et al. [15] and Abdou et al., nurses had moderate levels of MS [44]. In two other studies that were conducted on critical care nurses in China and Turkey, the level of MS was reported as moderate [8, 30]. A number of studies conducted in Iran also reported that nurses had moderate MS [20, 38, 45, 46]. MS enables nurses to provide effective ethical performance. Also, while making nurses sensitive to the moral issues faced in their professional work environment, MS enables nurses to make ethical decisions for the patient [47]. MS is affected by several factors, including upbringing, culture, religion, education, and experience, and is expressed differently by each person [48].

The present study showed a significant positive relationship between MS and SWB. Two other studies also showed a similar positive relationship between nurses and nursing students [49, 50]. SWB had a significant positive relationship with adherence to ethical codes. It was also a significant predictor of ethical performance, such that each unit increase in the total score of SWB led to an average increase of 0.15 units in the adherence to ethical code score (95% CI: 0.078–0.21). The results of a study in Iran showed a connection between SWB and ethical conduct [51]. The results of another study showed that spiritual intelligence has a significant relationship with adherence to ethical codes in nurses [52].

Spirituality has a close relationship with morality and is always considered morally and aesthetically valuable [53]. Organizations are becoming more and more interested in humanity, and the various dimensions of human beings, and, more importantly, spirituality, which is an inseparable part of human morality and values and has key significance in monotheistic religions. This importance is mostly due to the fact that human nature has a fundamental tendency toward positive values. Spirituality, spiritual growth, and its role in various matters of life have received more attention recently [51]. The review of theoretical principles and research background suggests that spirituality is linked to social responsibility and moral issues in organizations [21].

The results also showed that MS has a direct and significant relationship with adherence to ethical codes. MS has a predictive role in nurses' adherence to ethical codes, such that each unit increase in the total score of MS leads to an average increase of 0.31 units in the nurses' adherence to ethical codes score, and the MS score explains 7% of the variance in this moral performance score. This result agrees with the results of a study conducted to determine the relationship between MS and observing patients' rights in critical care units [26]. A study conducted by Ghasemi et al. (2018) also showed that ethical conduct has a significant positive relationship with SWB and MS [51]. MS is the first principle in personal morality that enables the subject's correct visualization of ethical performance in the face of ethical dilemmas. MS is necessary for ethical performance and enables nurses to interpret the patients' needs and respond to them according to ethical principles. It seems that Ms is related to high caring behavior in intensive care nurses, as mentioned in Taylan et al.'s study [54], even though the relationship between MS and ethical conduct is not always predictable [15].

In the study by Ghasemi et al., although the multivariate regression analysis carried out showed that MS and spiritual health predicted 4.9% of the variance in behavior, SWB had a greater share in predicting ethical conduct [51]. Meanwhile, in the present study, moral sensitivity had a greater effect on ICU nurses' adherence to ethical codes compared to spiritual health.

The strengths of the present study include its multicenter setting and measuring the adherence to ethical codes score according to the nurses' self-reports and evaluation by the head nurses. This study was conducted on nurses working in public hospitals, which limits the generalization of the results to private centers. It is suggested to sample nongovernment hospitals in future studies.

This study examined the relationship between very few variables in critical care units only; therefore, future studies are recommended to investigate the relationship between a larger number of variables and nurses' adherence to ethical codes and assess other clinical wards as well.

## 5. Conclusion

The results showed that adherence to ethical codes has a significant positive correlation with MS and SWB. Since nurses' ethical performance, especially in critical care units, is highly important, it can be concluded that reinforcing their SWB and MS improves their ethical performance. Measures should therefore be taken by managers to provide the necessary training to improve SWB and MS in nurses so that their adherence to ethical codes can also be improved. Through continuous education programs using learner-oriented strategies and based on reflection on real cases, it is possible to increase nurses' moral sensitivity. Also, spiritual counseling can be effective in improving their spiritual well-being. On the other hand in the recruitment and employment of nursing personnel, nursing managers may consider SW and MS as predictors of their moral performance. Moreover, considering the results of this study, nursing educators should place more stress on developing SW and MS in their students, especially through the hidden curriculum.

## Figures and Tables

**Table 1:** Mean score of critical nurses' adherence to ethical codes with respect to demographic characteristics.

Variables		N (%)	Mean ± SD	*P* value
Sex	Male	71	85.69 ± 9.89	0.47^*∗*^
	Female	227	86 ± 10.37	

Marital status	Single	179	86.61 ± 10.31	0.75^*∗*^
	Married	119	86.22 ± 10.21	

Education level	Bachelor	285	86.62 ± 10.32	0.18^*∗*^
	Master	13	82.76 ± 7.95	

Number of children	None	236	86.64 ± 10.27	0.36^*∗∗*^
	1	35	84.46 ± 10.06	
	≥2	27	85.39 ± 10.84	

^*∗*^ *t*-test; ^*∗∗*^ANOVA test.

**Table 2:** Pearson correlation between adherence to ethical codes and spiritual well-being among critical care nurses.

	Spiritual well-being	Religious health	Existential health
Adherence to ethical codes	*r* : 0.25	*r* = 0.18	*r* : 0.28
	*P* > 0.001	*P*=0.002	*P* > 0.001

**Table 3:** Pearson correlation between mean score adherence to ethical codes and moral sensitivity among critical care nurses.

Moral sensitivity	Correlation coefficient	*P* value
Using moral notions	0.19	0.001
Benevolence	0.12	0.034
Respect for patient's autonomy	0.26	*P* < 0.001
Knowing patient communication skills	0.06	0.29
Adherence to the law	0.21	*P* < 0.001
Moral conflict experience	0.13-	0.03
Moral sensitivity total score	0.27	*P* < 0.001

**Table 4:** The correlation among adherence to ethical codes, spiritual well-being, and moral sensitivity based on multiple regression analysis.

Variable	Unstandardized coefficients	Standardized coefficients	*R* Square	*R*	Sig
	*B*	*ß*			
Spiritual well-being	0.094	0.157	0.093	0.31	0.01
Moral sensitivity	0.162	0.21			0.001

## Data Availability

The data supporting the findings of this study are available from the corresponding author upon reasonable request.
